# Real world safety of CT-P10 (anti-CD 20 monoclonal antibodies biosimilar) in rheumatic and autoimmune diseases

**DOI:** 10.1186/s41927-022-00306-7

**Published:** 2022-11-30

**Authors:** Wanruchada Katchamart, Pintip Ngamjanyaporn, Annop Orawongpaisarn, Thossapoom Phubangkertphon, Sasimon Borrirukwisitsak, Nichapa Dechapaphapitak

**Affiliations:** 1 Division of Rheumatology, Department of Medicine, Faculty of Medicine Siriraj Hospital, Mahidol University, 2 Wanglang Road, Bangkoknoi, Bangkok, 10700 Thailand; 2 Division of Rheumatology, Department of Medicine, Faculty of Medicine Ramathibodi Hospital, Mahidol University, Bangkok, Thailand; 3 Department of Medicine, Faculty of Medicine Siriraj Hospital, Mahidol University, Bangkok, Thailand

**Keywords:** Rituximab, CT-P10, Biosimilar anti-CD 20 antibodies, Adverse event, Infusion reaction

## Abstract

**Background:**

Rituximab (RTX), anti-CD 20 monoclonal antibodies, has been approved for many rheumatic and autoimmune diseases, the use of RTX is still limited due to financial constrain. Biosimilar RTX may increase access by offering patients a more affordable option, lead to improved patient outcomes. However, real-world data related to its immediate and short-term safety is scarce. This study aimed to evaluate the real-world immediate and short-term safety profiles of CT-P10, a biosimilar of Rituximab, in patients with rheumatic and autoimmune diseases.

**Methods:**

This prospective study included patients diagnosed with rheumatic or autoimmune diseases, aged ≥ 18 years, who were treated with biosimilar RTX at Siriraj or Ramathibodi Hospital during February 2019 to May 2019. Patients were followed up through 6 months after the infusions.

**Results:**

Of the 74 patients, with 124 infusions, 84% were females with mean age (SD) of 49.4 (15.7) years. The most common rheumatic and autoimmune disease included in this study was systemic lupus erythematosus (26%). All immediate adverse events (AEs) (15 out of 124 infusions) were mild requiring only symptomatic and supportive treatment. Short-term AEs included infection (N = 35), hematologic abnormalities (N = 33), chylous ascites (N = 1), and others (N = 10). Two deaths were related to serious bacterial and viral infection. Hematologic AEs comprised anemia (N = 5), neutropenia (N = 10), lymphopenia (N = 15), and thrombocytopenia (N = 3).

**Conclusion:**

In this real-world study, biosimilar RTX (CT-P10) has favorable immediate and short-term safety profiles. However, further studies with large sample size and long-term follow-up in real-world practice are still required to confirm the result.

**Supplementary Information:**

The online version contains supplementary material available at 10.1186/s41927-022-00306-7.

## Introduction

Rituximab (RTX), anti-CD 20 monoclonal antibodies, is a widely used biologic agent for treatment of rheumatic and autoimmune diseases [1], including rheumatoid arthritis (RA), systemic lupus erythematosus (SLE), pemphigus vulgaris (PV), idiopathic inflammatory myopathy (IIM), and anti-neutrophil cytoplasmic antibody (ANCA)-associated vasculitis. Furthermore, other indications for rituximab in non-rheumatic and autoimmune diseases are chronic lymphocytic leukemia and non-Hodgkin lymphoma [1]. A systemic review [2] demonstrated its efficacy and safety profiles of RTX in combination with methotrexate over placebo plus methotrexate for the treatment of refractory RA, while its adverse events are comparable to those in placebo group. Its adverse events comprise infusion reactions, infections [3], and hematologic abnormalities, such as neutropenia [4]. Most adverse events are mild to moderate and associated with first infusion. Although RTX has been approved for many rheumatic and autoimmune diseases, the use of RTX is still limited. Many factors constraining patient access to RTX for the treatment of rheumatic and autoimmune diseases included restrictive treatment guidelines, administrative hurdles, and financial constraint. The availability of biosimilars may reduce barriers to access by offering patients a more affordable option, lead to improved patient outcomes.

CT-P10 (Truxima®) is a biosimilar of RTX approved by European Medicines Agency and Thai Food and Drug Administration for follicular lymphoma, diffuse large B cell non-Hodgkin’s lymphoma, chronic lymphocytic leukemia, RA, granulomatosis with polyangiitis, and pemphigus vulgaris. Based on the totality of evidence, both in vitro studies and clinical trials suggested that CT-P10 is highly similar to its reference product in terms of chemical structure, purity, biological activity, safety, efficacy, and immunogenicity in patients with RA. Nonetheless, real-world data related to its safety is scarce. The aim of this study was to evaluate the real-world immediate and short-term safety profiles of CT-P10 in patients with rheumatic and autoimmune diseases.

## Methods

This prospective study included patients who were diagnosed with rheumatic or autoimmune diseases at age of 18 years or older and received CT-P10 at Siriraj hospital and Ramathibodi hospital, Mahidol University, Bangkok. Thailand between February 2019 and May 2019. Written informed consent was obtained from all participants prior to enrollment.

Demographic data, baseline characteristics, and clinical data related to CT-P10 infusion were collected at baseline and 6 months after infusions for occurrences of adverse reactions. Adverse events (AEs) were stratified into immediate and short-term AE. Immediate AE was defined as any reactions that occurred within 24 h after infusion, include infusion reactions and anaphylaxis. Short-term AE was defined as any adverse events that occurred at 24 h until 6 months after infusion, including hematologic abnormality, elevated liver enzyme, infections, as well as other AEs. Severity of AEs was graded based on the Common Terminology Criteria for Adverse Events (CTCAE) Version 5 [5] (Additional file 1: Table S1). The causal relationship of AE was adjusted and documented based on the WHO-UMC Causality Categories [6] (Additional file 1: Table S2).

This study was approved by the Siriraj Institutional Review Boards (COA no. Si 671/2018) and Ramathibodi Institutional Review Boards (COA no. MURA2019/124). All procedures were in accordance with the ethical standards of the institutional and/or national research committee and with the 1964 Helsinki Declaration and its later amendments or comparable ethical standards. Informed consent was obtained from all individual participant prior to enrolling in the study.

All statistical analyses were performed using IBM SPSS Statistics for Windows, version 20.0 (IBM Corp., Armonk, N.Y., USA). For the quantitative data, descriptive statistics are expressed as mean ± standard deviation (SD) for normal distributed data, or median (IQR) values for non-normal distributed data. For the qualitative data, number and percentage (%) are reported. To compare patients with and without infection-related AE, continuous data were analysed using the independent *t*-test or the Mann–Whitney *U*-test, while proportions were analysed using X^2^ test or Fisher-exact tests, as appropriate.

## Results

A total of 74 patients, receiving 124 infusions were included in this analysis. Of these, 83.8% were females with mean age (SD) of 49.4 (15.7) years. The most common rheumatic and autoimmune disease was SLE (25.7%), followed by RA (24.3%), adult-onset immunodeficiency with anti-interferon gamma autoantibody (12.2%), and pemphigus vulgaris (10.8%). Prednisolone was the most common concomitant medication (68.9%), followed by antimalarials (41.9%), and methotrexate (27%) (Table 1). Indication for RTX were inadequate response to standard treatment (92%), experienced adverse events related to standard treatment (30%), and physicians’ discretion based on disease severity (2.7%). One-third (37.8%) were previously treated with RTX prior to enrollment, 21.6% RTX-biosimilar and 16.2% RTX-originator. The main reason for switching from originator to biosimilar was financial problem (98%).

**Table 1:** Baseline characteristics of study population (N = 74 cases)

Baseline characteristics	N (%) or mean ± SD
Age	49.38 ± 15.70
Female	62 (83.8)
Underlying disease	
Rheumatoid arthritis	16 (21.6)
Systemic lupus erythematosus	18 (24.3)
Adult-onset immunodeficiency with anti-IFN-gamma autoantibody	9 (12.2)
Pemphigus vulgaris	8 (10.8)
Sjogren’s syndrome	5 (6.8)
Inflammatory myopathy	5 (6.8)
IgG4-related disease	2 (2.7)
Focal segmental glomerulosclerosis	1 (1.4)
Systemic sclerosis	2 (2.7)
Neuromyelitis optica	1 (1.4)
Granulomatosis with polyangiitis	5 (6.8)
Overlapping syndrome	2 (2.7)
RA + SLE	1 (1.4)
RA + Sjogren’s syndrome	1 (1.4)
Concomitant medications	
Prednisolone	51 (68.9)
Antimalarial agent	31 (41.9)
Methotrexate	20 (27.0)
Azathioprine	16 (21.6)
Cyclophosphamide	16 (21.6)
Cyclosporine	8 (10.8)
Mycophenolate mofetil	10 (13.5)
Leflunomide	10 (13.5)
Sulfasalazine	5 (6.8)
Chlorambucil	2 (2.7)
Dexamethasone	2 (2.7)
Tacrolimus	1 (1.4)
Intravenous immunoglobulin	1 (1.4)

IFN, interferon gamma; IgG, immunoglobulin; RA, rheumatoid arthritis; SLE, systemic lupus erythematosus

### Immediate adverse events

There was 15 immediate AEs, including 6 infusion reactions, 5 skin reactions, and 4 respiratory symptoms (Table 2). All immediate AEs were mild requiring only symptomatic and supportive treatment, including temporary infusion interruption, and treatment with anti-histamine and short-course glucocorticoid. Neither severe infusion reaction nor anaphylaxis was found in this study.

**Table 2:** Immediate adverse events in 124 infusions

Adverse events (N = 15 events)	Number of events (%)
Infusion reaction	
Grade 1	4 (3.2)
Grade 2	2 (1.6)
Grade 3	0 (0.0)
Grade 4	0 (0.0)
Grade 5	0 (0.0)
Skin	
Rash	0 (0.0)
Flushing	4 (3.2)
Itching	1 (0.8)
Mucosa (angioedema)	0 (0.0)
Respiratory	
Shortness of breath	4 (3.2)
Wheeze	0 (0.0)
Stridor	0 (0.0)
Cough	0 (0.0)
Hypoxemia	0 (0.0)

### Short-term adverse events

Regarding short-term AEs (Table 3), there were 35 events related to infection, 33 hematologic abnormalities, 10 other AEs, and 2 deaths. Causes of death of all patients were related to severe infection.

**Table 3:** Long-term adverse events in 124 infusions

Events	Number of events (%)					
	Total	Grade 1	Grade 2	Grade 3	Grade 4	Grade 5
Infection (N = 35 events)						
Bacteria	14 (11.3)	1 (0.8)	2 (1.6)	8 (6)	1 (0.8)	2 (1.6)
*Pseudomonas* spp. septicemia	4 (3.2)	0 (0.0)	0 (0.0)	2 (1.6)	1 (0.8)	1 (0.8)
*E.coli* septicemia	3 (2.4)	0 (0.0)	0 (0.0)	2 (1.6)	0 (0.0)	1 (0.8)
Bacterial infection/diarrhea	4 (3.2)	1 (0.8)	2 (1.6)	1 (0.8)	0 (0.0)	0 (0.0)
Acute bacterial cholangitis	2 (1.6)	0 (0.0)	0 (0.0)	2 (1.6)	0 (0.0)	0 (0.0)
Gram negative septicemia	1 (0.8)	0 (0.0)	0 (0.0)	1 (0.8)	0 (0.0)	0 (0.0)
Virus	15 (12.1)	13 (10.5)	0 (0.0)	2 (1.6)	0 (0.0)	0 (0.0)
URI	10 (8.1)	10 (8.1)	0 (0.0)	0 (0.0)	0 (0.0)	0 (0.0)
Viral gastroenteritis	1 (0.8)	1 (0.8)	0 (0.0)	0 (0.0)	0 (0.0)	0 (0.0)
Varicella	1 (0.8)	1 (0.8)	0 (0.0)	0 (0.0)	0 (0.0)	0 (0.0)
Herpes viral infection	1 (0.8)	1 (0.8)	0 (0.0)	0 (0.0)	0 (0.0)	0 (0.0)
CMV infection	2 (1.6)	0 (0.0)	0 (0.0)	2 (1.6)	0 (0.0)	0 (0.0)
Fungus	6 (4.8)	0 (0.0)	1 (0.8)	3 (2.4)	2 (1.6)	0 (0.0)
Pneumonia	3 (2.4)	0 (0.0)	0 (0.0)	3 (2.4)	0 (0.0)	0 (0.0)
IPA with LUL aneurysm rupture	2 (1.6)	0 (0.0)	0 (0.0)	0 (0.0)	2 (1.6)	0 (0.0)
Cutaneous candidiasis	1 (0.8)	0 (0.0)	1 (0.8)	0 (0.0)	0 (0.0)	0 (0.0)
Mycobacteria	2 (1.6)	0 (0.0)	0 (0.0)	2 (1.6)	0 (0.0)	0 (0.0)
Hematologic (N = 33 events)						
Anemia	5 (4.0)	2 (1.6)	2 (1.6)	1 (0.8)	0 (0.0)	0 (0.0)
Neutropenia	10 (8.1)	0 (0.0)	6 (4.8)	4 (3.2)	0 (0.0)	0 (0.0)
Lymphopenia	15 (12.1)	8 (6.5)	7 (6.5)	0 (0.0)	0 (0.0)	0 (0.0)
Thrombocytopenia	3 (2.4)	1 (0.8)	1 (0.8)	1 (0.8)	0 (0.0)	0 (0.0)
Chylous ascites (N = 1 event)	1 (0.8)	0 (0.0)	0 (0.0)	1 (0.8)	0 (0.0)	0 (0.0)
Others (N = 10 events)						
Transaminitis	6 (4.8)	6 (4.8)	0 (0.0)	0 (0.0)	0 (0.0)	0 (0.0)
Respiratory failure	1 (0.8)	1 (0.8)	0 (0.0)	0 (0.0)	0 (0.0)	0 (0.0)
Muscle pain	1 (0.8)	1 (0.8)	0 (0.0)	0 (0.0)	0 (0.0)	0 (0.0)
Fatigue	1 (0.8)	0 (0.0)	1 (0.8)	0 (0.0)	0 (0.0)	0 (0.0)
Nausea/Vomiting	1 (0.8)	0 (0.0)	0 (0.0)	1 (0.8)	0 (0.0)	0 (0.0)

URI, upper respiratory tract viral infection; CMV, cytomegalovirus; IPA, invasive pulmonary aspergillosis; LUL, left upper lung aneurysm

For infection, viral infection was the most common AE, 10 episodes of upper respiratory tract infection, 2 episodes of cytomegalovirus (CMV) viremia, 1 episode of gastroenteritis, 1 episode of varicella infection, and 1 episode of herpes infection. Most viral infections (87%) were mild to moderate, except a 49-year-old woman who was diagnosed of systemic sclerosis with nonspecific interstitial pneumonitis, active myopathy, and neuropathy developed CMV pneumonia and finally died of respiratory failure 20 days after the first dose of 1000 mg of CT-P10 infusion. Bacterial infections occurred 14 episodes (4 episodes of diarrhea and pseudomonas septicemia each, 3 episodes of *E. coli* septicemia, 2 episodes of acute cholangitis, 1 episode of gram-negative septicemia). One patient with pemphigus vulgaris died of severe pneumonia caused by *Pseudomonas aeruginosa*, and *Enterococcus* spp., which was 10 days after the first dose of 500 mg of CT-P 10 infusion. For fungal infections, there were 3 episodes of pneumocystis pneumonia, 2 episodes of invasive pulmonary aspergillosis (IPA), and 1 episode of cutaneous candidiasis. One patient with IPA developed ruptured aneurysm at left upper lung. Two episodes of mycobacterial infection were caused by *mycobacterium avium complex* and *non-tuberculous mycobacterium* spp.

For hematologic abnormalities, anemia, neutropenia, lymphopenia, and thrombocytopenia were found in 5, 10, 15, and 3 episodes, respectively*.* However, all of them were asymptomatic.

Other uncommon short-term AEs comprised elevated liver enzymes (n = 6), chylous ascites (n = 1), myalgia (n = 1), fatigue (n = 1), and nausea/vomiting (n = 1). All these AEs were mild to moderate requiring no treatment.

There was no difference between age, sex, underlying rheumatic or autoimmune diseases, steroid use, immunosuppressive agent or disease modifying antirheumatic drug use, or cytopenia between patients with or without bacterial, viral, and fungal infections (Additional file 1: Tables S3–S5).

## Discussion

While a systematic review and meta-analysis [2] reported that 29–38% of patients with RA developed immediate AEs during the 1st infusion of RTX originator, we found only15 immediate AEs out of 124 infusions (12%) from RTX biosimilar in this study. Although the discrepancy may be caused by a small sample size in this study, this available data suggested that the immediate AEs related to CT-P10 infusion was acceptable.

From recent systematic review and meta-analysis [7] in which aimed to assess the difference in rate of infections between biologic RTX and non-RTX treatment in patients with RA, there was no significant difference between biologic RTX and non-RTX groups in overall infections rate (43.3% vs 44.9%; odds ratio [OR] = 0.87; 95% CI 0.70–1.08). Subgroup analysis also showed no significant differences in overall infections rate between biologic RTX versus placebo or other immunosuppressive agents, so an incidence of infections from RTX was suspected to be confounded by these concomitant medications.

Similar to previous report of RTX originator in real-world databases, hematologic abnormalities was the most common AE, mostly were lymphopenia, followed by neutropenia [8, 9]. For lymphopenia, this also directly related to the mechanism of action of anti-CD20 monoclonal antibodies, which is B-cell depletion leading to attenuating immune dysregulation in rheumatic and autoimmune diseases. Patients with lymphopenia are susceptible to viral infections, such as cytomegalovirus, herpes, and varicella as shown in our study.

For neutropenia, the recent French registry, called “AutoImmunity and Rituximab (AIR) registry” [8], reported the prevalence of neutropenia in RA and autoimmune diseases were 1.3% and 2.3%, respectively. Median time after infusion to onset of neutropenia was 102 (40–362) days [9], resulted in 7 hospitalizations. In our study, prevalence of neutropenia in RA and autoimmune diseases was similar to previous study (less than 5%). Only 1 of 9 patients with bacterial, fungal, or mycobacterial infection had neutropenia.

Regarding elevated liver enzymes, chylous ascites, and constitutional symptoms, we still had limited information related to these adverse events. Elevation of liver enzymes related to RTX infusions was reported in previous study [10] in patients with chronic lymphocytic leukemia who received RTX originator leading to a recommendation of closely monitoring liver enzymes after RTX treatment. From recent case-report [11] of patient with overlap syndrome of mixed connective tissue disease and SLE with chylous ascites, there was an excellent resolution of chylous ascites after biologic RTX infusion, so we suspected that the chylous ascites may be incidentally found in our study and not be associated with biosimilar RTX. We still need more information to interpret these findings.

Some limitations need to be concerned in this study. First, the number of patients included in this study was quite low, consequently uncommon or rare adverse events may not be discovered. Second, the diversity of underlying rheumatic or autoimmune diseases in this study may lead to various incidence of adverse events due to disease itself or concomitant medications for those conditions. Lastly, there was no control group with RTX originator in this study. Therefore, the AE-related to RTX biosimilar relative to RTX originator is still unclear and merits exploration.

Despite all these limitations, our study showed that CT-P10, biosimilar RTX, has favorable immediate and short-term safety profiles in real-world. However, further pharmacovigilance studies with large sample size and long-term follow up are still required to confirm its safety.

## Supplementary Information

**Additional file 1: Table S1.** Definitions of severity (based on the Common Terminology Criteria for Adverse Events (CTCAE) Version 5). **Table S2.** WHO-UMC causality categories. **Table S3.** Factors associated with bacterial infection (N = 74). **Table S4.** Factors associated with viral infection (N = 74). **Table S5.** Factors associated with fungal infection (N = 74).

## Data Availability

The datasets used and/or analysed during the current study are available from the corresponding author on reasonable request.
