# Supplementary material for: Real world safety of CT-P10 (anti-CD 20 monoclonal antibodies biosimilar) in rheumatic and autoimmune diseases
Source: BMC Rheumatol. 2022 Nov 30;6:77. doi: 10.1186/s41927-022-00306-7 (PMC9710159; doi:10.1186/s41927-022-00306-7)
Supplement: Supplementary file 1 — Additional file 1: Table S1. Definitions of severity (based on the Common Terminology Criteria for Adverse Events (CTCAE) Version 5). Table S2. WHO-UMC causality categories. Table S3. Factors associated with bacterial infection (N = 74). Table S4. Factors associated with viral infection (N = 74). Table S5. Factors associated with fungal infection (N = 74). [file 41927_2022_306_MOESM1_ESM.docx]

**Additional file 1: Table S1.** Definitions of severity (based on the Common Terminology Criteria for Adverse Events (CTCAE) Version 5)

| **Type of adverse event** | **Definition** |
| --- | --- |
| **Hematologic abnormalities**  • Anemia  Grade 1  Grade 2  Grade 3  Grade 4  Grade 5  • Neutropenia  Grade 1  Grade 2  Grade 3  Grade 4  Grade 5  • Lymphopenia  Grade 1  Grade 2  Grade 3  Grade 4  Grade 5  • Thrombocytopenia  Grade 1  Grade 2  Grade 3  Grade 4  Grade 5 | Hb < LLN to 10 g/dL  Hb < 10 g/dL to 8 g/dL  Hb < 8 g/dL; transfusion indicated  Life-threatening consequences; urgent intervention indicated  Death  < LLN to 1,500/mm^3^  < 1,500 to 1,000/mm3  < 1,000 to 500/mm3  < 500/mm3  Death  < LLN to 800/mm3  < 800 to 500/mm3  < 500 to 200/mm3  < 200/mm3  Death  < LLL to 75,000/mm3  < 75,000 to 50,000/mm3  < 50,000 to 25,000/mm3  < 25,000/mm3  Death |
| **Infection**  Grade 1  Grade 2  Grade 3  Grade 4  Grade 5 | Asymptomatic or mild symptoms; clinical or diagnostic observations only; intervention not indicated  Localized; local intervention indicated (e.g. topical antibiotic, antifungal, or antiviral)  IV antibiotic, antifungal, or antiviral intervention indicated; invasive intervention indicated  Life-threatening consequences; urgent intervention indicated  Death |
| **Other adverse events**  Grade 1  Grade 2  Grade 3  Grade 4  Grade 5 | Mild  Moderate  Severe  Life-threatening; urgent intervention indicated  Death related to AE |

**Additional file 1: Table S2.** WHO-UMC Causality Categories

| **Causality term assessment criteria** |
| --- |
| **Certain**  • Event or laboratory test abnormality, with plausible time relationship to drug intake  • Cannot be explained by disease or other drugs  • Response to withdrawal plausible (pharmacologically, pathologically)  • Event definitive pharmacologically or phenomenologically (i.e. an objective and specific medical disorder or a recognized pharmacological phenomenon)  • Re-challenge satisfactory, if necessary |
| **Probable /Likely**  • Event or laboratory test abnormality, with reasonable time relationship to drug intake  • Unlikely to be attributed to disease or other drugs  • Response to withdrawal clinically reasonable  • Re-challenge not required |
| **Possible**  • Event or laboratory test abnormality, with reasonable time relationship to drug intake  • Could also be explained by disease or other drugs  • Information on drug withdrawal may be lacking or unclear |
| **Unlikely**  • Event or laboratory test abnormality, with a time to drug intake that makes a relationship improbable (but not impossible)  • Disease or other drugs provide plausible explanations |
| **Conditional /Unclassified**  • Event or laboratory test abnormality  • More data for proper assessment needed, or  • Additional data under examination |
| **Unassessable/Unclassifiable**  • Report suggesting an adverse reaction  • Cannot be judged because information is insufficient |

**Additional file 1: Table S3.** Factors associated with bacterial infection (N=74)

| Factors | Patients WITH  Bacterial infections | Patients WITHOUT Bacterial infections | P value |
| --- | --- | --- | --- |
| All cases | 8(10.8) | 66(89.2) |  |
| Age | 46.9±20.6 | 49.7±15.2 | 0.634 |
| Female | 5(62.5) | 57(86.4) | 0.115 |
| RA vs. Non-RA (Other diseases) | 1(12.5) | 17(25.8) | 0.670 |
| Steroid use* | 6(75.0) | 47(71.2) | 1.00 |
| Immunosuppressive agent use** | 4(50.0) | 54(81.8) | 0.061 |
| Neutropenia | 0(0.0) | 4(6.1) | 1.00 |
| Lymphopenia | 2(25.0) | 9(13.6) | 0.339 |

* Prednisolone, dexamethasone, Methyprednisolone

**Methotrexate, Azathioprine, Cyclosporine A, Mycophenolate mofetil, Leflunomide, Cyclophosphamide, Sulfasalazine, Chlorambucil, Traculimus

**Additional file 1: Table S4.** Factors associated with viral infection (N=74)

| Factors | Patients WITH  viral infections | Patients WITHOUT viral infections | P value |
| --- | --- | --- | --- |
| All cases | 8(10.8) | 66(89.2) |  |
| Age | 42.4±11.9 | 50.2±16.0 | 0.186 |
| Female | 7(87.5) | 55(83.3) | 1.00 |
| RA vs. Non-RA (Other diseases) | 2(25.0) | 16(24.2) | 1.00 |
| Steroid use* | 6(75.0) | 47(71.2) | 1.00 |
| Immunosuppressive agent use** | 7(87.5) | 51(77.3) | 0.678 |
| Neutropenia | 1(12.5) | 3(4.5) | 0.374 |
| Lymphopenia | 2(25.0) | 9(13.6) | 0.339 |

* Prednisolone, dexamethasone, Methyprednisolone

**Methotrexate, Azathioprine, Cyclosporine A, Mycophenolate mofetil, Leflunomide, Cyclophosphamide, Sulfasalazine, Chlorambucil, Traculimus

**Additional file 1: Table S5.** Factors associated with fungal infection (N=74)

| Factors | Patients WITH  fungal infections | Patients WITHOUT fungal infections | P value |
| --- | --- | --- | --- |
| All cases | 3(4.1) | 71(95.9) |  |
| Age | 49.7±24.7 | 49.4±15.5 | 0.968 |
| Female | 2(66.7) | 60(84.5) | 0.417 |
| RA vs. Non-RA (Other diseases) | 1(33.3) | 17(23.9) | 1.00 |
| Steroid use* | 2(66.7) | 51(71.8) | 1.00 |
| Immunosuppressive agent use** | 2(66.7) | 56(78.9) | 0.524 |
| Neutropenia | 0(0.0) | 4(5.6) | 1.00 |
| Lymphopenia | 1(33.3) | 10(14.1) | 0.387 |

* Prednisolone, dexamethasone, Methyprednisolone

**Methotrexate, Azathioprine, Cyclosporine A, Mycophenolate mofetil, Leflunomide, Cyclophosphamide, Sulfasalazine, Chlorambucil, Traculimus
